# ECG analysis of ventricular fibrillation dynamics reflects ischaemic progression subject to variability in patient anatomy and electrode location

**DOI:** 10.3389/fcvm.2024.1408822

**Published:** 2024-11-27

**Authors:** Hector Martinez-Navarro, Ambre Bertrand, Ruben Doste, Hannah Smith, Jakub Tomek, Giuseppe Ristagno, Rafael S. Oliveira, Rodrigo Weber dos Santos, Sandeep V. Pandit, Blanca Rodriguez

**Affiliations:** ^1^ Department of Computer Science, British Heart Foundation Centre of Research Excellence, University of Oxford, Oxford, United Kingdom; ^2^ Department of Physiology, Anatomy, and Genetics, University of Oxford, Oxford, United Kingdom; ^3^ Dipartimento di Fisiopatologia Medico-Chirurgica e dei Trapianti, Università degli Studi di Milano Statale, Milano, Italy; ^4^ Computer Science Department, Universidade Federal de São João del Rei, São João del Rei, Brazil; ^5^ Departamento de Ciência da Computação, Universidade Federal de Juiz de Fora, Juiz de Fora, Brazil; ^6^ Scientific Affairs, ZOLL Medical Corporation, Chelmsford, MA, United States

**Keywords:** cardiac electrophysiology, modelling and simulation, ventricular fibrillation, myocardial ischaemia, arrhythmia, electrocardiogram

## Abstract

**Background:**

Ventricular fibrillation (VF) is the deadliest arrhythmia, often caused by myocardial ischaemia. VF patients require urgent intervention planned quickly and non-invasively. However, the accuracy with which electrocardiographic (ECG) markers reflect the underlying arrhythmic substrate is unknown.

**Methods:**

We analysed how ECG metrics reflect the fibrillatory dynamics of electrical excitation and ischaemic substrate. For this, we developed a human-based computational modelling and simulation framework for the quantification of ECG metrics, namely, frequency, slope, and amplitude spectrum area (AMSA) during VF in acute ischaemia for several electrode configurations. Simulations reproduced experimental and clinical findings in 21 scenarios presenting variability in the location and transmural extent of regional ischaemia, and severity of ischaemia in the remote myocardium secondary to VF.

**Results:**

Regional acute myocardial ischaemia facilitated re-entries, potentially breaking up into VF. Ischaemia in the remote myocardium modulated fibrillation dynamics. Cases presenting a mildly ischaemic remote myocardium yielded sustained VF, enabled by the high proliferation of phase singularities (PS, 11–22) causing remarkably disorganised activation patterns. Conversely, global acute ischaemia induced stable rotors (3–12 PS). Changes in frequency and morphology of the ECG during VF reproduced clinical findings but did not show a direct correlation with the underlying wave dynamics. AMSA allowed the precise stratification of VF according to ischaemic severity in the remote myocardium (healthy: 23.62–24.45 mV Hz; mild ischaemia: 10.58–21.47 mV Hz; moderate ischaemia: 4.82–11.12 mV Hz). Within the context of clinical reference values, apex-anterior and apex-posterior electrode configurations were the most discriminatory in stratifying VF based on the underlying ischaemic substrate.

**Conclusion:**

This *in silico* study provides further insights into non-invasive patient-specific strategies for assessing acute ventricular arrhythmias. The use of reliable ECG markers to characterise VF is critical for developing tailored resuscitation strategies.

## 1 Introduction

Ischaemic heart disease is the leading cause of death worldwide (1, 2). Myocardial ischaemia arises from a mismatch between the supply and consumption of oxygen and nutrients, and poor waste removal, often due to the narrowing of a coronary artery. The first 10–15 min of acute myocardial ischaemia, or phase 1A, are particularly pro-arrhythmic due to increased heterogeneity of repolarisation and conduction properties around the ischaemic region (3). Electrophysiological heterogeneities establish the pro-arrhythmic substrate for re-entrant waves, tachycardia, and potentially ventricular fibrillation (VF) and cardiac arrest (4, 5).

Myocardial ischaemia is both a precursor and a consequence of VF. Fibrillatory activity prevents the ventricles from pumping blood effectively, resulting in compromised myocardial perfusion, and thus secondary myocardial ischaemia in the remote myocardium. Clinical and simulation studies have shown that, during VF, the inherently progressive ischaemic substrate modulates the complexity of fibrillatory wavefronts in human hearts, which may impact treatment success (6). Thus, patients need urgent and effective treatment as early as 3 min from the VF onset (7, 8), and the non-invasive characterisation of VF dynamics is crucial for the optimisation of resuscitation protocols.

The electrocardiographic (ECG) signal is the only available diagnostic tool for the non-invasive characterisation of VF dynamics during resuscitation, an extremely time-pressing intervention. Real-time analysis of ECG signal in VF has the potential to predict VF termination and survival through a patient-specific defibrillation strategy (9–11). However, it is unclear how accurately the electrocardiographic signature of VF reflects fibrillation dynamics and to which extent the properties of the ischaemic substrate affect theś ECG signature of VF.

Fibrillation dynamics have been historically quantified by calculating the dominant frequency (DF) for a specific time window, both in human and animal studies (12, 13). Here, we assess the use of novel electrocardiographic markers, such as amplitude spectrum area (AMSA) and median slope (MS), which aim to achieve higher predictive accuracy than DF without relying on invasive and expensive methods. The complete transparency of simulation data also allows comparing ECG-based metrics with the complexity of fibrillatory dynamics quantified through phase singularities (PS) as also done experimentally using multi-electrode epicardial sock (6) or optical mapping (14).

The goal of this study was to quantify how ECG metrics (including AMSA, DF, and MS) reflect fibrillatory dynamics during myocardial ischaemia using human-based modelling and simulation, building on extensive experimental and clinical datasets for its development, calibration, and validation. Multiscale modelling and simulation have provided crucial insights into the mechanisms of VF (15, 16) and myocardial ischaemia (17–19). The technology has the advantage of allowing precise control of conditions imposed and transparent, high-resolution datasets. We hypothesise that specific ECG markers reflect not only VF wave dynamics but also the electrophysiological properties of the underlying ischaemic substrate, enabling time-sensitive guidance for VF intervention.

## 2 Methods

### 2.1 Human-based modelling and simulation framework for VF in ischaemia

**Figure 1:**
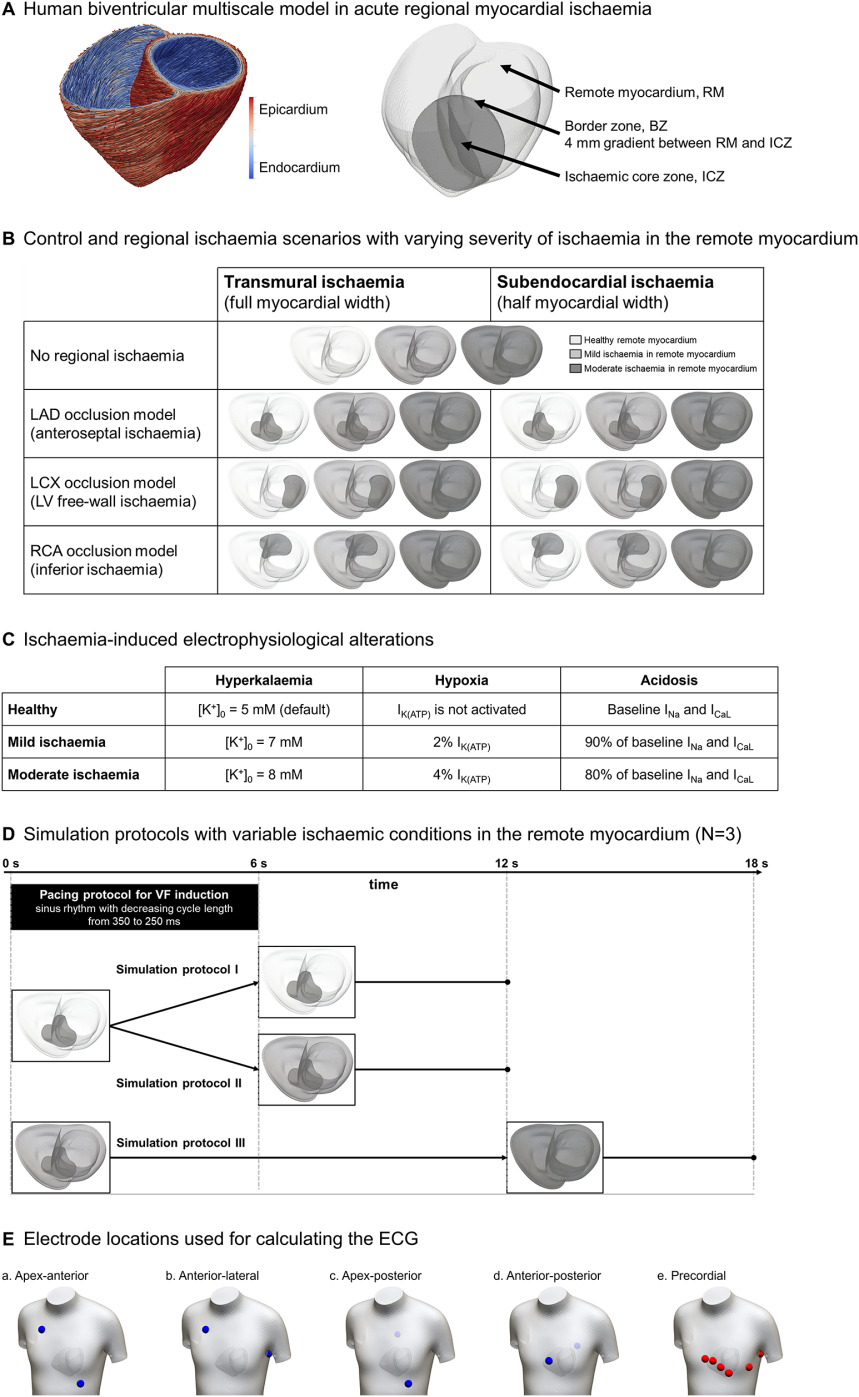
Human-based modelling and simulation framework for ischaemia-induced ventricular fibrillation. **(A)** Human biventricular model obtained from clinical magnetic resonance images, including rule-based fibre orientation (left) and ischaemia-induced electrophysiological alterations (right). **(B)** 21 scenarios were considered including control versus ischaemic regions in three locations (rows), 2 degrees of transmurality (columns), and 3 degrees of ischaemic severity in remote myocardium (colour-coded). **(C)** Degrees of ischaemic severity considered in the simulations; the top row represents healthy values for reference. Regional ischaemia is considered “moderate”. **(D)** Simulation protocols: (I) healthy remote myocardium (0–12 s); (II) healthy remote myocardium (0–6 s) becoming mildly ischaemic (6–12 s); (III) mildly ischaemic remote myocardium (0–12 s) becoming moderately ischaemic (12–18 s) leading to global ischaemia. **(E)** Electrode locations used for ECG simulation.

As in the studies by Martinez-Navarro et al. and Riebel et al. (17, 20), a biventricular model of human electrophysiology embedded in a torso was created through the integration of an anatomical model from clinical magnetic resonance images of a human subject, and extensive multiscale experimental and clinical data from ionic to body surface potentials. Myocardial fibre orientation was represented using a rule-based method (21) reproducing the experimental findings of Streeter et al. (22) (Figure 1A, left). Under healthy conditions, longitudinal conduction velocity along the fibre direction was 65 and 50 cm/s in the transversal and normal directions. The conductivities in the fast conductive endocardial layer were four times higher than in the rest of the ventricles to obtain realistic QRS morphologies and durations.

The human ventricular cell ToR-ORd model (23), with established credibility for healthy, disease, and drug block conditions, was used to represent membrane kinetics. Transmural heterogeneities were defined through a sensitivity analysis to obtain realistic T waves in the electrocardiogram: 50% endocardium, 30% mid-myocardium, and 20% epicardium cardiomyocytes (23). The experimentally reported apicobasal gradient (24) in the slow delayed rectifier potassium current (*I*_Ks_) was also introduced leading to gradients in action potential (AP) duration of 11 ms.

Electrophysiological effects of acute ischaemia, caused by hyperkalaemia, hypoxia, and acidosis, were incorporated into the ischaemic tissue to model the heterogeneous changes in refractoriness and conduction velocity in human ischaemic tissue (17, 25, 26). The formulation of the ATP-dependant potassium current *I*_K(ATP)_ (27) was incorporated to simulate hypoxic conditions. Electrophysiological alterations in the ischaemic zone were included as in Martinez-Navarro et al. and Dutta et al. (17, 25), considering the ischaemic core zone (ICZ) and the lateral border zone (BZ), which represents an electrophysiological gradient between the ICZ and the remote myocardium (Figure 1A, right). In addition, the subendocardial border zone described by Wilensky et al. (28) was included to consider oxygen diffusion from blood in the ventricular cavities.

### 2.2 Scenarios of regional ischaemia and VF

As illustrated in Figure 1B, our simulations included variability in the location of regional ischaemia—left anterior descending artery (LAD), left circumflex artery (LCX), and right coronary artery (RCA) occlusions, see rows in Figure 1B—transmural extent (transmural and subendocardial, see columns in Figure 1B), and ischaemic severity in the remote myocardium due to impaired myocardial circulation (grey scale: healthy tissue, *mild ischaemia* and *moderate ischaemia*, described in Figure 1C). ICZ severity was the same in all scenarios (*moderate ischaemia)*, corresponding to the first minutes post-occlusion, as seen in previous studies (17, 19, 26).

To evaluate the impact of ischaemia in the remote myocardium in fibrillatory dynamics, each of the seven scenarios was simulated under three simulation protocols (Figure 1D). In protocol I, 12 s of electrical activity are simulated with healthy remote myocardium. In protocol II, the remote myocardium transitions from healthy to mildly ischaemic after 6 s. Finally, in protocol III, the remote myocardium is mildly ischaemic for 12 s and becomes moderately ischaemic for 6 additional seconds (representing conditions of global myocardial ischaemia).

### 2.3 Stimulation protocols

Sinus rhythm was simulated to obtain realistic ECGs by implementing endocardial stimulation in root nodes (four in the left ventricle, three in the right ventricle) and a fast endocardial layer representing the Purkinje network (29, 30). VF was induced by a stimulation protocol with progressively higher pacing frequency. The state variables of the cellular models in the biventricular simulations were loaded from single-cell simulations with a cycle length (CL) of 350 ms, for each cell type and ischaemic severity. Then, two regular beats at CL = 350 ms were simulated, followed by a sequence of sinus rhythm stimuli with progressively shorter CL (3× CL = 325 ms, 5× CL = 300 ms, 5× CL = 275 ms, 6× CL = 250 ms), taking approximately 6 seconds—last stimulus set when *t* = 5,850 ms—to complete the pacing protocol (black rectangle in Figure 1D). No further pacing was induced until the end of the simulations to assess VF sustenance.

### 2.4 Quantification of electrocardiographic markers and wave dynamics

The ECG signal was computed in four bipolar electrode locations typically considered in external defibrillation protocols: (a) apex-anterior, (b) anterior-lateral, (c) apex-posterior, and (d) anterior-posterior electrodes, as shown in Figure 1E (31). In addition, the ECG was computed at standard clinical precordial lead locations in the 12-lead electrocardiogram (e).

ECG signal during VF was analysed in the frequency domain using the fast Fourier transform (FFT) to obtain the DF and AMSA (10, 19). AMSA was calculated as the sum of the products of each frequency and their amplitudes: AMSA = ∑*A_i_* · *F_i_*, where *A_i_* represents the amplitude at the *i*th frequency *F_i_*. The calculation parameters reproduced those in the study by Ristagno et al. (10): the signal was resampled at 250 Hz and analysed on a 512-point window (2.05 s), and a Tukey FFT window (*α* = 0.2) was used to minimise edge effects in the analysed window. An additional ECG marker was calculated, namely, the MS of the signal as in the study by Neurauter and Strohmenger (9). For consistency, DF and MS were measured on the same time windows as AMSA.

Wave dynamics throughout the ventricles in VF were quantified as in Martinez-Navarro et al. (17) by identifying the number of PS, calculated as the lines formed by overlapping two isosurfaces in the ventricles: *V_m_* = −40 mV and *dV_m_*/*dt* = −2. The number of PS was determined in the midpoint of the time windows used for electrocardiographic markers.

### 2.5 Optimisation of the human cell model for VF dynamics (ToR-ORd_VF_)

The original formulation of the ToR-ORd human cardiomyocyte model exhibits a relatively shallow action potential duration (APD) restitution curve and limited capability to trigger alternans, both important for fibrillation (32). Therefore, to optimise the model for VF investigations, we created a population of 1,000 variations of the ToR-ORd model using Latin Hypercube Sampling by varying all current conductances and the inactivation dynamics in currents critical for restitution dynamics (*I*_Na_, *I*_NaL_, *I*_CaL_, *I*_Kr_). Firstly, only those models (*N* = 475) reproducing experimental data in humans describing AP morphology and intracellular calcium measurements (33) were considered (Supplementary Figure S1). A second calibration step was performed to retain only the models that reproduced the AP duration ranges reported in Sutton's work (26) under ischaemia conditions (phase 1A) and a pacing frequency of 2 Hz (Supplementary Figure S2), resulting in 275 variations of the ToR-ORd model. The S1S2 restitution protocol was applied to these models, selecting the 17 variations with the steepest restitution curve. Finally, the dynamic restitution protocol was applied to these models (and baseline ToR-ORd) to investigate their capability to yield alternans. For this study, we chose the ToR-ORd version with the steepest AP restitution and greatest APD alternans amplitude (Supplementary Figures S3A,B, respectively) assuming this model would be the most prone to trigger spiral breakup and alternans. Only models presenting no repolarisation abnormalities at short CLs were considered valid (Supplementary Figure S4). The features of the selected model variant, ToR-ORd_VF_, are achieved by a combination of factors, including slower inactivation dynamics in the *I*_Na_ (50% decrease of *τ*_h_ and *τ*_j_) and *I*_NaL_ (34% decrease of *τ*_h,L_) currents, as well as increased conductance of the same currents (39% increase for both). These changes altered tissue propagation, leading to a 5% reduction in the myocardial conduction velocity. A complete characterisation of the model is provided in the Supplementary Methods (Supplementary Figure S5). In addition, tissue conductivities were reduced to yield conduction velocities ∼20% slower than in the healthy myocardium to promote arrhythmia (34, 35), accounting for a 25% conduction velocity reduction compared to the baseline model at tissue level.

### 2.6 Verification and validation

All simulations were conducted on the open-source solver MonoAlg3D (36) using graphics processing units (GPUs). For verification, a set of simulations computed in MonoAlg3D was verified against those obtained from other cardiac electrophysiology solvers, such as Chaste (30). All simulations produced identical ECG morphologies and those run on MonoAlg3D incurred lower computational costs and hardware requirements. Simulations were run in the supercomputers Piz Daint (Swiss National Supercomputing Centre), Cirrus (Edinburgh Parallel Computing Centre, UK), and Polaris (Argonne Leadership Computing Facility, USA). About 10 s of simulated human cardiac electrophysiology took ∼18 h, incurring a lower cost than simulations run on solvers that scale only on a multi-CPU paradigm (∼7× speedup).

Model credibility is supported by validation of the original human ventricular cell ToR-ORd model with extensive human data, as described by Tomek et al. (23) for healthy, diseased, and drug block conditions. In the new ToR-ORd_VF_ formulation, AP morphology and calcium dynamics both in healthy and ischaemia conditions reproduced experimental data in human (26, 33) while exhibiting alternans and a steeper AP restitution. Furthermore, simulations in MonoAlg3D with ToR-ORd_VF_ showed agreement with experiments in human tissue (37), and ECGs obtained with biventricular simulations were evaluated by comparing the simulated ECGs with clinical records as shown in the next section.

## 3 Results

### 3.1 Comparison of simulated ECG and VF dynamics with experimental and clinical data

**Figure 2:**
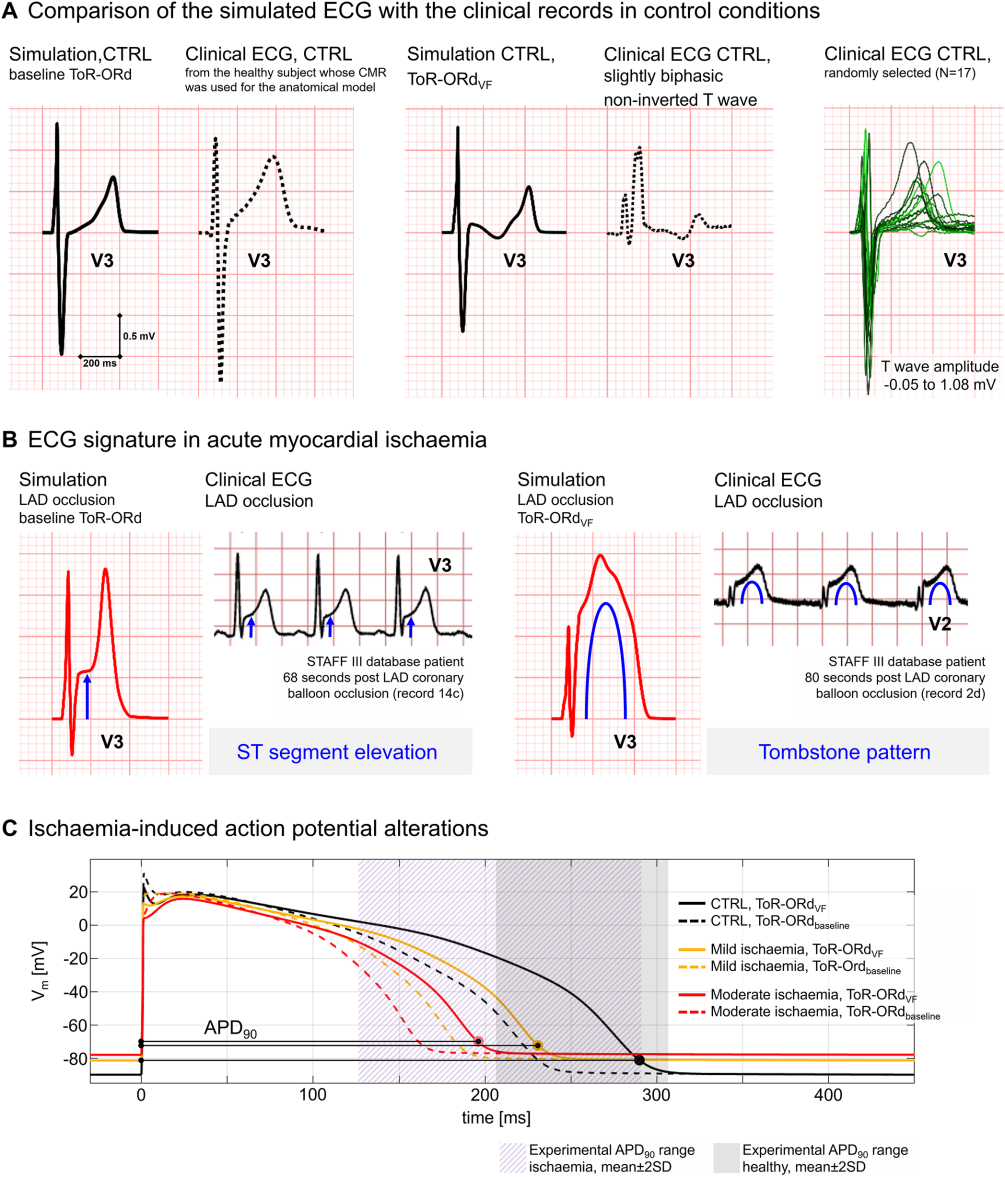
Model validation based on ECG and action potential (AP). **(A)** Comparison of simulated ECGs under healthy conditions with clinical ECGs from the healthy subjects. Left: Healthy simulation using the ToR-ORd model (solid line) vs. ECG record from the patient whose cardiac magnetic resonance images were used to construct the anatomical model (dashed line). Right: healthy simulation using the ToR-ORd_VF_ model (solid line) vs. a clinical ECG from a healthy subject presenting similar T wave morphology (dashed line, patient from the UK Biobank). **(B)** Comparison of the electrocardiographic alterations induced by acute ischaemia in simulated ECGs and representative clinical ECGs from the STAFF III database. In our simulations, ST elevation is obtained using the baseline ToR-ORd model (left panel), whereas the tombstone pattern was reproduced using the ToR-ORd_VF_ model (right panel). **(C)** Simulated AP with the ToR-ORd_VF_ (solid lines) and ToR-ORd baseline (dashed lines) models in healthy (black), mild ischaemia (orange), and moderate ischaemia (red) conditions. AP durations (APD90) are in agreement with the ranges of experimental data in human ventricular cells reported in Sutton et al. (26) in control conditions (purple area) and after 2–3 min of ischaemia (grey area).

Figure 2 highlights the agreement of simulated and clinical ECGs, supporting the credibility of simulations in healthy (Figure 2A) and ischaemic conditions (Figure 2B). Simulated ECGs using the baseline ToR-ORd and the optimised ToR-ORd_VF_ cell models show similar patterns to representative clinical ECGs from healthy patients obtained from the study by Mincholé et al. (30) and the UK Biobank (38). T waves in healthy patients show variable amplitudes and morphologies but are typically positive in V3 (0.3–0.6 mV), as shown in Figure 2A (right panel). Figure 2B shows how simulations using the baseline ToR-ORd model can reproduce the distinctive ECG signature of acute ischaemia (39, 40), such as hyperacute T waves and ST elevation ≥0.2 mV (left panel), as in our previous work (17). The simulated ECGs are compared with ECGs from ischaemic patients accessed via the PhysioNet repository (https://physionet.org/), specifically the STAFF database, collected with Institutional Review Board approval (40). Interestingly, the inclusion of the ToR-ORd_VF_ model characterised by a steep APD restitution (see Section 2.5 for full model description), allowed us to reproduce more complex ischaemia-induced ECG abnormalities in our simulations. Specifically, we were able to reproduce the tombstone pattern, characterised by an elevated ST segment merged with the T wave, larger than the preceding R wave, which tends to be short and of small amplitude (41) (Figure 2B, right panel). This pattern has been observed in certain anterior wall ischaemia/infarction patients (42) and is linked with higher mortality (43).

To further support model credibility at the cellular level, Figure 2C illustrates that both the baseline ToR-ORd and ToR-ORd_VF_ models (dashed and solid lines, respectively) yield APD_90_ within experimental ranges in control (grey region) and ischaemic conditions (purple region) (26). Simulations with ToR-ORd_VF_ produce a steeper and realistic (44, 45) restitution curve (maximal steepness of 0.6 when DI > 280 ms, 3.7 when DI < 280 ms) and increased capability to reproduce alternans while showing no repolarisation abnormalities at physiological pacing frequencies. More information is provided in the Supplementary Methods section.

**Figure 3:**
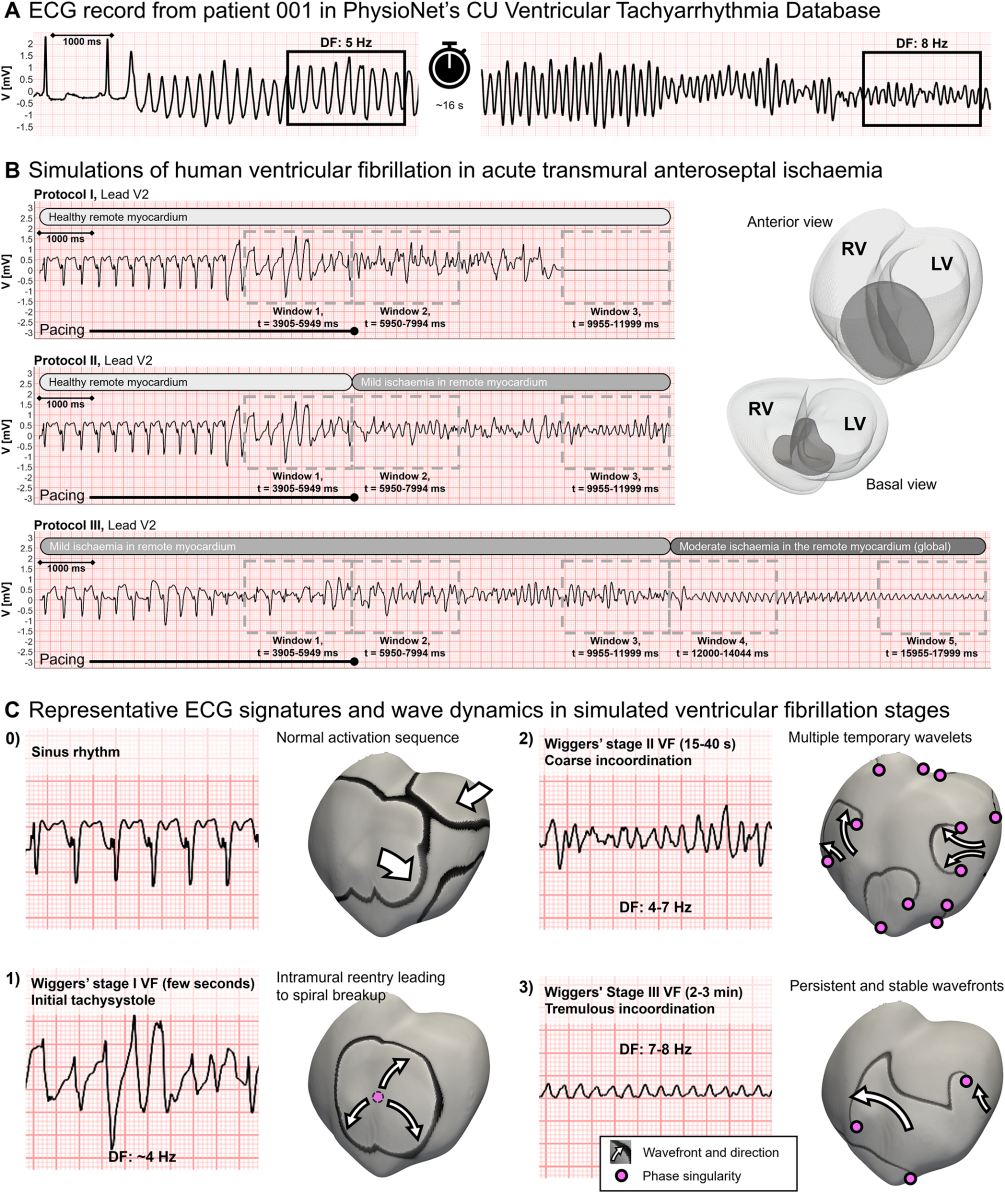
Comparison of clinical and simulated ECGs in ventricular fibrillation (VF). **(A)** Clinical ECG recording during VF from PhysioNet database. Dominant frequencies (DF) were annotated 3 and 28 seconds post-VF onset. **(B)** Simulated ECGs during VF in acute regional ischaemia following LAD coronary artery occlusion (right panel, darker region depicts the ischaemic core zone) during simulation protocols (i), (ii), and (iii) (top, middle, and bottom panels, respectively). ECG markers and wavefronts were quantified in windows of 2,045 ms. **(C)** Typical ECG signature, wavefront analysis, and phase singularities in the different VF stages in the simulations: panel 0 represents sinus rhythm, whereas panels 1, 2, and 3 correlate to Wiggers' VF stages I, II, and III, respectively, as reported in their seminal work (48). For each phase, the range of typical DF values is provided.

Simulated ECGs in VF simulations were compared with clinical recordings from the PhysioNet repository (https://physionet.org/), and specifically the Creighton University Ventricular Tachyarrhythmia Database (46, 47), collected with Institutional Review Board approval. Figure 3A shows a representative clinical ECG recording from a patient during a VF episode: after sinus rhythm is replaced by an arrhythmia, DF accelerates progressively from 5 to 8 Hz; 24 seconds after arrhythmia onset, signal amplitude decreases from 2 to 1 mV. These changes, happening within a minute from VF onset, suggest a continuously changing substrate caused by secondary acute myocardial ischaemia (48). Figure 3B presents simulated ECGs during anteroseptal ischaemia leading to VF. We simulated the first few minutes of cardiac activity after arrhythmia onset by increasing the severity of the ischaemic substrate, represented as grey horizontal bars. As a result, we observed similar changes in DF and signal amplitude to those in the clinical record.

For further comparison with clinical and experimental data, we quantified VF dynamics and electrocardiographic markers in five time windows across the simulations for ventricular tachycardia/fibrillation (VT/VF) onset (Figure 3B, window 1, *t* = 3,905–5,950 ms), VF persistence (Figure 3B, windows 2 and 3, *t* = 5,950–7,995 ms and *t* = 9,955–12,000 ms, respectively), and transition to global ischaemic conditions (Figure 3B, windows 4 and 5, *t* = 12,000–14,045 ms and *t* = 15,955–18,000 ms, respectively).

Figure 3C further highlights the agreement of simulated dynamics with reported ECG and activation patterns extracted for the classic VF stages defined by Wiggers (48). Wavefronts are shown as black bands over a grey background and PS as magenta circles.

• Sinus rhythm. Simulation of normal myocardial activation at an increasingly fast pacing frequency (CL = 350–250 ms) to induce VF. The ECG presents ST elevation, typically associated with acute myocardial ischaemia.
• *Initial tachysystole*, corresponding to Wiggers' stage 1. This stage lasts 1–2 seconds and represents the transition from sinus rhythm to VF. The underlying mechanism is one single re-entry sustained for a few cycles before breaking into wavelets. In the simulation shown, in transmural ischaemia by LAD occlusion, the intramural re-entry is enabled by the ischaemic border zone in the septum. ECG waves are irregular but show an amplitude and frequency similar to a fast-paced sinus rhythm (∼4 Hz).
• Multiple unstable wavelets, referred to as *coarse incoordination* by Wiggers. After the spiral breakup, multiple wavelets interact with each other in irregular patterns. The creation of new wavefronts or cancellation of existing ones is usual. The irregular ECG waves oscillate in significantly higher frequencies, in the range of 3–9 Hz. This pattern was reproduced when ischaemia in the remote myocardium was mild or inexistent, and spontaneous defibrillation may happen by wave cancellation.
• Persistent spiral waves, referred to as *tremulous incoordination* by Wiggers. This VF stage is characterised by a reduction in the number of PS and a very regular electrocardiographic signature with a DF of 7–8 Hz. This stage occurred when ischaemia in the remote myocardium increases to moderate, reproducing global ischaemia conditions. VF at this stage did not self-terminate as wave cancellations rarely occurred.

### 3.2 Acute myocardial ischaemia in the remote myocardium promotes and sustains VF

**Figure 4:**
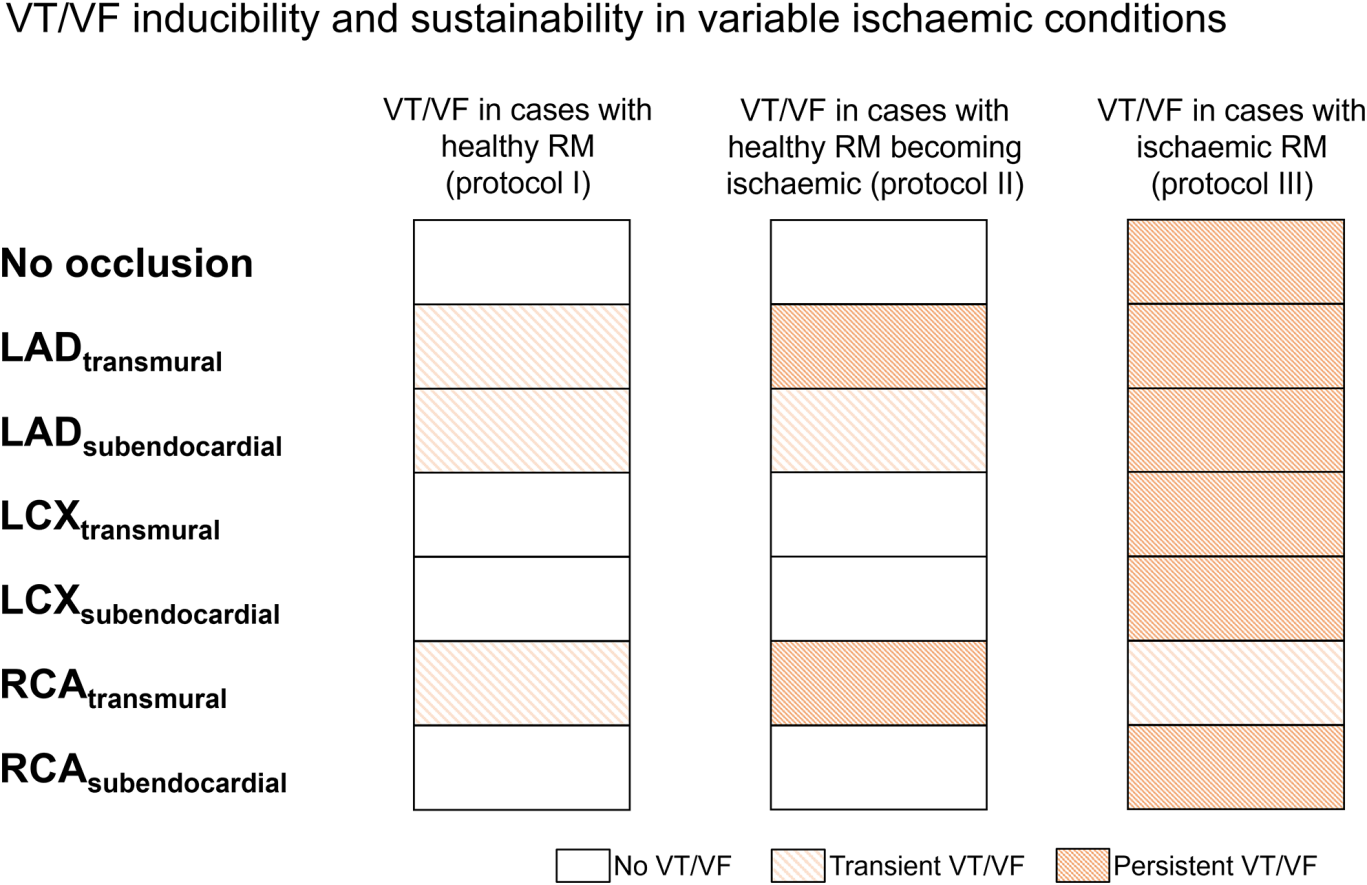
Ischaemic remote myocardium promoted ventricular tachycardia/fibrillation (VT/VF) inducibility and persistence in regional ischaemia simulations. For each regional ischaemia scenario (rows), the presence of VT/VF was quantified with healthy (left), healthy progressing to ischaemic after VT/VF induction (centre), and ischaemic (right) remote myocardium. White cells represent no arrhythmia, whereas light and dark-coloured cells indicate transient and persistent VT/VF, respectively; LAD, left anterior descending artery; LCX, left circumflex artery; RCA, right coronary artery.

We quantified arrhythmia inducibility for the different regional ischaemia configurations for healthy versus ischaemic remote myocardium. As shown in Figure 4, three out of seven cases featuring a healthy remote myocardium (left column, simulation protocol I) led to transient VT/VF episodes, as they self-terminated within the 12 seconds simulated. Out of these three VT/VF cases, two of them led to persistent VF if ischaemia was introduced in the remote myocardium after VF induction (centre column, simulation protocol II). Finally, VT/VF occurred in all scenarios with ischaemic remote myocardium, and six out of seven cases presented persistent VF throughout the whole simulation (right column, simulation protocol III).

### 3.3 ECG analysis enables reliable stratification of VF cases according to ischaemic severity

**Figure 5:**
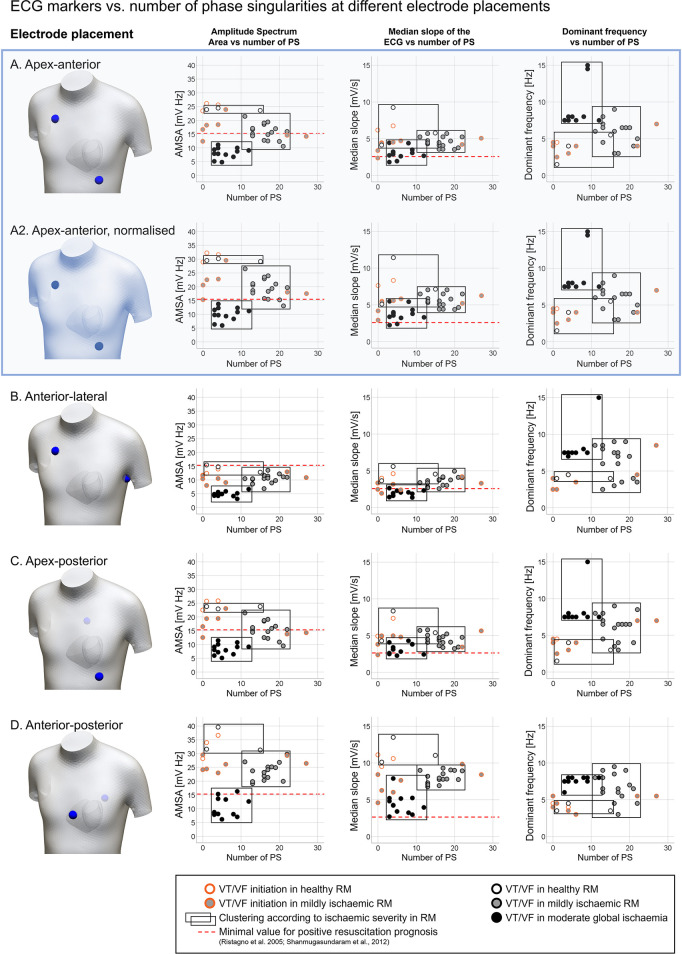
Quantification of ECG markers (amplitude spectrum area (AMSA); median slope (MS); dominant frequency (DF)) vs. number of phase singularities (PS) detected, considering multiple bipolar electrode configurations typically used in resuscitation protocols. **(A)** Apex-anterior, (**A2**) apex-anterior, normalised signal (max amplitude of 1 mV during sinus rhythm), **(B)** anterior-lateral, **(C)** apex-posterior, and **(D)** anterior-posterior. Measurements were obtained from ventricular tachycardia/fibrillation (VT/VF) time windows under variable ischaemic conditions, represented by the symbol colour: white, regional ischaemia + healthy remote myocardium (RM); grey, regional ischaemia + mild ischaemia in RM; and black, global moderate ischaemia. Cases considering the VT/VF onset (orange border) were excluded in the clustering according to ischaemic severity (black rectangles). Dashed red lines represent the minimal values associated with favourable prognosis in the literature (10, 49).

Figure 5 illustrates the manifestation of VF dynamics in the ECG through three ECG-extracted metrics (AMSA, MS, DF) for five electrode configurations compared to PS. The electrode configurations considered are commonly used in resuscitation protocols: apex-anterior (Figure 5A), normalised apex-anterior for 1 mV amplitude (Figure 5A2), anterior-lateral (Figure 5B), apex-posterior (Figure 5C), and anterior-posterior (Figure 5D) locations.

Each panel includes a graphical representation of the electrode placement, and plots of ECG markers—AMSA, MS, and DF in the left, centre, and right columns, respectively—vs. the number of PS. Circles in the scatter plots represent a measurement from the simulations. The colour of the circles codes the degree of ischaemia in the remote myocardium: white (regional ischaemia + healthy remote myocardium), grey (regional ischaemia + mildly ischaemic remote myocardium), and black (global moderate ischaemia). Data points are visually clustered in black rectangles to represent the value ranges measured for each ischaemic configuration. Measurements obtained from the VT/VF onset stage (circles with orange border) were excluded in the clustering, due to the high disparity in the number of PS and ECG marker values shown during spiral breakup. The red dashed line in AMSA and MS plots shows the minimal threshold values linked with positive patient outcomes in defibrillation (10, 49) to provide the context within reference clinical values to our simulation results.

The number of PS was consistent among panels in Figure 5 as the cases considered for each electrode configuration were the same. VF in cases with a healthy remote myocardium (white points) was uncommon, with only three data points excluding VT/VF onset measurements (1, 4, and 15 PS, white circles). The number of PS stabilises in the range of 11–22 in cases with a mildly ischaemic remote myocardium (grey circles) and 3–12 in global moderate ischaemia (black circles), in line with experimental human measurements (6).

AMSA, MS, and DF showed differences in value ranges depending on both ischaemic conditions and the ECG electrode placement. Apex-anterior electrodes (Figure 5A) were considered representative of the different electrode configurations. AMSA values (Figure 5A, left scatter plot) varied according to the severity of ischaemia in the remote myocardium (healthy: 23.62–24.45 mV Hz; mild ischaemia: 10.58–21.47 mV Hz; moderate global ischaemia: 4.82–11.12 mV Hz), allowing the stratification of VF cases according to ischaemic conditions. Hence, the overlap in AMSA value ranges is minor, represented graphically by the black rectangles.

MS values also decreased as the severity of ischaemia increased, but there was substantial overlap in the value ranges observed across different ischaemic conditions (Figure 5A, centre scatter plot; healthy: 4.13–9.25 mV/s; mild ischaemia: 3.54–5.71 mV/s; moderate global ischaemia: 1.82–4.44 mV/s). Regardless of ischaemic severity, stratification remained challenging around the threshold of MS = 4.2 mV/s. The red dashed line represents the clinical reference value from the study by Shanmugasundaram et al. (49) (2.6 mV/s). Finally, DF values (Figure 5A, right scatter plot) also varied according to the severity of ischaemia in the remote myocardium. In the healthy case, DF oscillates from normal rhythm to tachycardia values (1.5–5.5 Hz). A wide range of DF values was observed in mild ischaemia (3.0–9.0 Hz) but global moderate ischaemia stabilised DF to 7.5–8.0 Hz (except for cases reporting the second harmonic of that frequency, ∼15 Hz).

Subsequently, AMSA was characterised as the most suitable ECG marker for stratifying VF according to the underlying ischaemic substrate. The different electrode locations (Figures 5A–D) showed variability in their discriminatory value. Simulations using apex-anterior (Figure 5A) and apex-posterior (Figure 5C) electrode configurations characterised global myocardial ischaemia cases with AMSA values below the reference value linked to poor resuscitation prognostic, 15.5 mV Hz as in the study by Ristagno et al. (10) (red dashed line). However, the stratification of VF based on the ischaemic substrate in other electrode locations would require different threshold values for AMSA.

While the range of DF values was consistent across electrode locations (1.5–15 Hz), the range of AMSA and MS values changed based on the electrode configuration used. We hypothesised that this change could be due to the variability in the ECG signal amplitude. To confirm this, we compared ECG markers extracted from the same electrode configuration, with and without normalisation. Figure 5A shows ECG markers based on the original simulated ECG signal, and Figure 5A2 presents measurements from the normalised simulated ECG signal (amplitude of 1 mV during VF), which required scaling up the original signal by 23%. DF values remained unaltered post-normalisation (right plot in Figures 5A,B), whereas AMSA and MS measurements increased proportionally to the signal amplitude (left and centre plots, Figure 5A,A2). Even though AMSA can stratify VF cases according to the underlying ischaemic substrate, assessment based uniquely on clinical reference values may not suffice, as the ECG signal amplitude shifts the range of AMSA values adopted.

### 3.4 Progressive ischaemic severity in remote myocardium facilitates first spiral breakup and then VF stabilisation

**Figure 6:**
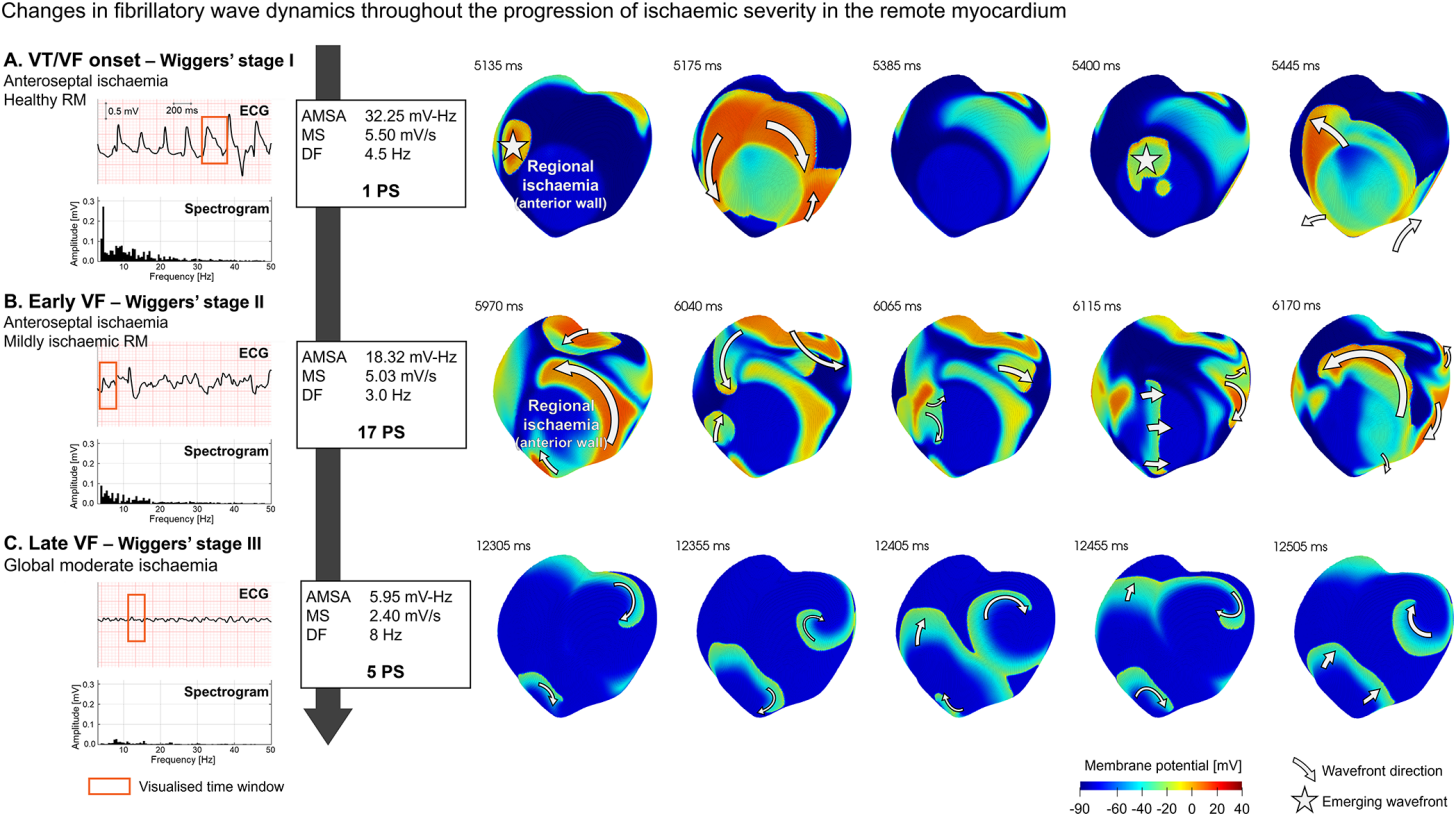
Progression of ventricular fibrillation (VF) wave dynamics and ECG signature in transmural regional ischaemia (LAD occlusion) at increasing ischaemic severity in the remote myocardium (RM): **(A)** VT/VF onset, **(B)** early VF, and **(C)** late VF. ECG and spectrogram of the signal at different stages with annotations for amplitude spectrum area (AMSA), median slope (MS), dominant frequency (DF), and the number of phase singularities (PS) (left panel). Membrane potential maps (right panel) for selected time intervals of the ECG (red rectangle) to illustrate representative wave dynamics at that VF stage. Emerging subendocardial wavefronts are identified with a star, while wavefronts in the epicardial surfaces show direction annotations shaped as arrows. ECG marker values are based on apex-anterior electrode configuration.

As shown in Figure 5, ECG biomarkers and number of PS vary with the progression of ischaemia in the remote myocardium, reflecting changes in VF complexity. To provide further mechanistic insights, Figure 6 illustrates this progression through different VF stages:

A. VT/VF onset with healthy remote myocardium (Figure 6A), corresponding to re-entrant patterns with a low range of PS. A single re-entry (1 PS) shown in Figure 6A was sustained through the ischaemic BZ in the septum, forming a re-entrant pathway emerging periodically at the epicardium (5,135–5,400 ms). This activation pattern is reflected in the ECG by a tachycardia-like signature featuring periodic and organised whole-ventricular depolarisations, leading to elevated AMSA (>15.5 mV Hz) and MS values (>2.6 mV/s), linked with a positive patient resuscitation outcome.
B. Early VF with mild ischaemia (Figure 6B), corresponding to complex re-entrant patterns with an increased number of PS (17 PS). The spiral wave break led to multiple unstable wavelets, producing a highly irregular activation pattern. Interestingly, the frequency spectrogram of the ECG still showed a wide range of frequencies but a smaller contribution of the low frequencies, leading to lower AMSA values.
C. Late VF in global moderate ischaemia (Figure 6C), corresponding to stable fibrillatory dynamics. Sustained VF is supported on a regular number of PS (7 PS). Stable rotors anchored to their location facilitated sustained spiral waves with reduced membrane potential. DF increased to expected values in human VF (∼8 Hz) and MS decreased severely (2.40 mV/s). The spectrum of frequencies present on the ECG signal was very limited; only frequencies adjacent to the DF were represented. The homogenisation of frequencies on the ECG signal was well represented by a drastic decrease in AMSA values compared to previous phases (82% AMSA reduction from VF onset).

Supplementary Video S1 shows a representative example of the VT/VF progression described above.

As shown in Figure 6A and reported extensively in the literature (17, 50), the ischaemic BZ in regional ischaemia provides a powerful substrate for re-entrant wavefronts. In our simulations, regional ischaemia induced the necessary electrophysiological heterogeneities for re-entries leading to tachycardia-like ECG signatures in cases with healthy remote myocardium (see Figure 6A). However, spiral breakup and posterior progression of VF depended on the presence of secondary ischaemia in the remote myocardium. Supplementary Figure S6A shows a VF simulation under global myocardial ischaemia conditions. The membrane potential maps in Panel A represent the fibrillatory wave dynamics (*t* = 11,760–12,160 ms) at the transition from global mild to moderate ischaemia (first and second row, respectively). The highly disorganised activation pattern based on multiple unstable wavelets (13 PS) quickly evolved into a lower number of very stable rotors (9 PS), and the ECG signature changed accordingly (Supplementary Figure S6B, AMSA dropped from 18.60 to 10.79 mV-Hz). The red rectangle represents the time window visualised in Panel A, and the increase in ischaemic severity is annotated by the blue vertical line. These results suggest that ischaemic severity determined alterations in VF wave dynamics and ECG signature, while heterogeneities caused by regional ischaemia were responsible for the occurrence of re-entrant pathways, potentially leading to arrhythmias.

## 4 Discussion

In this study, we provide quantification of ECG markers and VF complexity during acute ischaemia considering five ECG electrode configurations, analysed through the precise control enabled by human multiscale ventricular modelling and simulations. Simulations of VT/VF were conducted in 21 clinically relevant scenarios in acute ischaemia, considering variability in location, transmural extent, and severity of ischaemia. Comparison to experimental and clinical recordings from single cell to ECG, in sinus rhythm and VT/VF provides credibility to our findings. Our results identify a specific ECG-based metric, AMSA, as the most sensitive marker reflecting the evolution of the severity of the ischaemic substrate during VF. Electrode configurations apex-anterior and apex-posterior seem to provide better discrimination for ischaemic severity and VF dynamics than anterior-lateral and anterior-posterior. In terms of VT/VF dynamics, simulations also show how arrhythmia onset is followed by spiral wave break facilitated by the steep restitution properties and mild ischaemia in the remote myocardium. Further progression in ischaemic severity during VT/VF led to the stabilisation of rotors and hence sustained VF.

The validation of the human ventricular model with experimental and clinical ECGs allowed us to reproduce human cardiac electrophysiology accurately both in acute myocardial ischaemia and healthy conditions, from cell level to body surface potentials. We considered variable scenarios of acute regional ischaemia co-existing with different ischaemic severities in the remote myocardium: healthy, mildly ischaemic, and moderately ischaemic (global ischaemia). Some cases with healthy remote myocardium led to transient self-terminating VF episodes, while ischaemia in the remote myocardium was a strong inductor of persistent VF. Anteroseptal regional ischaemia was found to be particularly pro-arrhythmic, in agreement with previous clinical and computational studies (17, 51). Our VF simulations show progressive changes in the ECG and wave dynamics, characterised by reduced ECG amplitudes, increased fibrillatory frequencies, and higher myocardial activation incoordination, as reported in clinical recordings (6, 52). Clinically and experimentally reported stages within the first few minutes of VF were recognisable in our simulations, characterised by their wave dynamics and electrocardiographic signature (6, 48).

While the ischaemic region is key to the establishment of re-entry, ischaemic severity in the remote myocardium was critical for the spiral breakup and posterior fibrillation dynamics. Cases with a healthy remote myocardium were able to initiate ventricular arrhythmias through re-entrant pathways established by regional ischaemia (17, 50). Low inducibility and time duration of VF episodes in these conditions are coherent with the low number of PS. In contrast, in cases with mild ischaemia in the remote myocardium, the steep APD restitution and the mild electrophysiological remodelling cause proliferation of re-entrant waves and phase singularities (19, 32, 53), which increases the risk of spiral breakup dramatically. This behaviour is preserved before more severe ischaemic remodelling occurs (54). Under these conditions, the generated wavefronts varied rapidly as they cancelled each other. Conversely, global moderate ischaemia led to stable VF dynamics fuelled by persistent wavefronts anchored to a consistent number of PS, as seen in the study by Bradley et al. (6). This is justified by the flatter APD restitution and steeper excitation restitution in more severe ischaemic conditions (53), in addition to the impaired electrical propagation (37). Despite the chaotic electrical activation observed in the simulations of this fibrillation stage, fibrillatory wavefronts were characterised by their stability, potentially a major obstacle in VF termination in patients undergoing unsuccessful cardiopulmonary resuscitation for a prolonged time (55).

Healthy remote myocardium cases presented relatively low DF (<5 Hz) and very irregular ECG waveforms, whereas mildly ischaemic remote myocardium cases were linked with higher frequencies on the ECG (DF = 3–9 Hz) caused by the spiral breakup and subsequent numerous wavelets. Finally, global moderate ischaemia cases showed very stable low-amplitude (∼0.3 mV) high-frequency (DF = ∼8 Hz) ECG waves in VF, as reported experimentally (56, 57) and clinically (52). These electrocardiographic features are consistent with “fine VF”, typical after 5–10 min of VF onset. Under these conditions, the ECG signal is composed of a progressively narrower range of frequencies, centred around the DF. This marker showed great agreement with experimental values in human VF (4–9 Hz) (6, 58) but ranges were unspecific, and inferring information on the underlying ischaemic substrate would be merely speculative.

To quantify wave dynamics, we calculated the number of PS in each scenario. Typically, this method in clinical and experimental studies requires invasive procedures, such as epicardial socks (6) or optical mapping (14). Even though the distribution of PS in the myocardium provides high-resolution spatiotemporal information on wave dynamics, the number of rotors in early VF was very volatile and oscillated widely in the first few seconds. Interestingly, we observed that the pro-arrhythmic substrate was characterised not only by the specific number of PS at a given timestamp but also its variability. Thus, exploiting the ECG signature of VF may provide a more informative insight into whole-organ fibrillation dynamics, circumventing the use of invasive techniques.

In the first minutes of simulated VF, we observed a substantial decrease in the amplitude of the signal and an increase in DF, in agreement with experimental values published in other studies on human investigating the dynamics of VF after onset (6, 19, 56). Bradley et al. (6) showed that the number of PS increases substantially in the first 30 s after VF onset from 5.5 to 7.7 PS (mean values) and maintains stability for the next 90 s. The VF simulations reproduced also clear PS proliferation from VF onset (single re-entry) to global ischaemia (3–12 PS), a range consistent with human experimental data. However, our simulations identify a transition period from VT/VF onset to global ischaemia conditions corresponding with a spiral wave breakup, characterised by a high complexity (11–22 PS), not seen in the work by Bradley et al. (6). The authors hypothesise that this observation could be caused by technical limitations in the epicardial sock measurements in patients since the quantification of PS is restricted by the resolution of the acquisition methodology and the region considered (only epicardium). Alternatively, the absence of sinus rhythm stimulation in the simulations after VF onset may reproduce more complex VF dynamics than in patients, perhaps due to their still-functioning ventricular conduction system propagating sinus rhythm.

Our VF simulations also reproduce the clinical range of values adopted by AMSA and their monotonic decrease during ischaemic progression (10, 11). We have provided mechanistic evidence supporting AMSA as a quantitative marker of the ischaemic substrate by linking wave dynamics to the ECG signature. AMSA has previously been proposed as a novel, non-invasive method to estimate resuscitation success, and reported better performance than coronary perfusion pressure, considered the gold standard even though the technique relies on patient catheterisation (59). In our simulations, AMSA values dropped consistently after VF onset, both in early VF—right after spiral wave breakup into wavelets—and late VF, when the arrhythmia is sustained by a lower number of stable rotors. AMSA was the most accurate ECG marker describing the changes in the ischaemic substrate, the main modulator of VF stability. Thus, the heterogeneity of frequencies on the ECG was a better tool to assess VF dynamics than other features, both novel—such as the median slope of the signal—and traditional waveform measurements (59), or the number of PS, which varies rapidly.

Finally, while suggesting that AMSA is a powerful ECG marker to quantify the VF substrate, our study also showed that AMSA values vary depending on the electrode configuration. This variability is not currently considered in AMSA-based VF stratification, where clinical threshold values do not account for electrode positioning. AMSA values were highest in the anterior-posterior electrode configuration, in which one lead is placed over the sternum (V2 in the 12-lead ECG); configurations including one lead placed below the left breast (heart apex, relatively close to V5 in the 12-lead ECG) presented lower values, but still higher than the anterior-lateral configuration, in which one lead is placed in the left axilla (V6 in the 12-lead ECG). These findings are fully consistent with the study conducted by Thannhauser et al. (60), who identified lead-dependent differences in AMSA ranges as an impediment to the accurate categorisation of VF. These results strongly support the need to carefully consider the electrode placement in a real-life resuscitation setting.

While the DF remained unchanged by alterations in ECG signal amplitude, AMSA measurements changed proportionally. ECG signal amplitude had a great impact on AMSA ranges, hence representing an important confounder in VF characterisation. This effect could be exacerbated in patients with very high or very low BMI, as electric damping properties of body fat may alter AMSA values and thus their associated prognosis. Even though AMSA allows an accurate stratification of VF according to the ischaemic substrate independently of the signal amplitude, the lack of consistency in the range of AMSA values obtained could affect the interpretability of the marker in the context of clinical reference values. Therefore, it is safe to establish that anatomical factors, such as electrode placement and the amount of adipose tissue in the patient's torso, may act as confounders if clinical decisions are taken based strictly on absolute AMSA values.

## 5 Limitations

Our human ventricular simulations incorporate a phenomenological model of the His–Purkinje network to reproduce a realistic activation sequence. The potential role of the Purkinje network in modulating fibrillatory dynamics is therefore not considered. However, VF characteristics in our simulations are consistent with experimental and clinical recordings, supporting their credibility. Further computational studies could focus on optimising or personalising current VF assessment techniques, which would require the creation of large and heterogeneous populations of virtual scenarios allowing the generalisation of the findings or the development of new metrics considering potential confounders imposed by patient variability. Future studies could also consider the effects of cardiopulmonary resuscitation on VF, by enabling a partial myocardial perfusion in the remote myocardium.

## 6 Conclusion

This study provides a detailed quantification of VF dynamics in an ischaemic setting by ECG markers, accounting for variability in location, transmural extent, and severity of ischaemia. We show that AMSA is the most sensitive ECG-derived marker of the ischaemic substrate during VF but is highly affected by signal amplitude and electrode location. Apex-anterior and apex-posterior electrode locations provide better discrimination of ischaemic severity when contextualised with clinical reference values. These findings highlight the importance of considering patient chest anatomy and ECG electrode positioning when performing AMSA-based resuscitation protocols. Further computational and clinical studies are needed to explore new techniques or algorithms for improved VF assessment which account for these factors.

## Data Availability

The original contributions presented in the study are publicly available. These data and the code required to replicate them can be found in the InSiFib dataset (61) (https://zenodo.org/records/10818689).
