# Supplementary material for: ECG analysis of ventricular fibrillation dynamics reflects ischaemic progression subject to variability in patient anatomy and electrode location
Source: Front Cardiovasc Med. 2024 Nov 27;11:1408822. doi: 10.3389/fcvm.2024.1408822 (PMC11631900; doi:10.3389/fcvm.2024.1408822)
Supplement: Supplementary file 2 [file Datasheet1.docx]

Supplementary Material

# Development of the ToR-ORd_VF_ model

A population of models (PoM) based on 1000 variations of the ToR-ORd model of human ventricular action potential (AP) was created using Latin Hypercube Sampling to explore the parameter space in the 21 parameters, namely the conductances of every ionic current in the model and the inactivation/activation time constants in selected currents (I_Na_, I_NaL_, I_CaL_, I_Kr_).


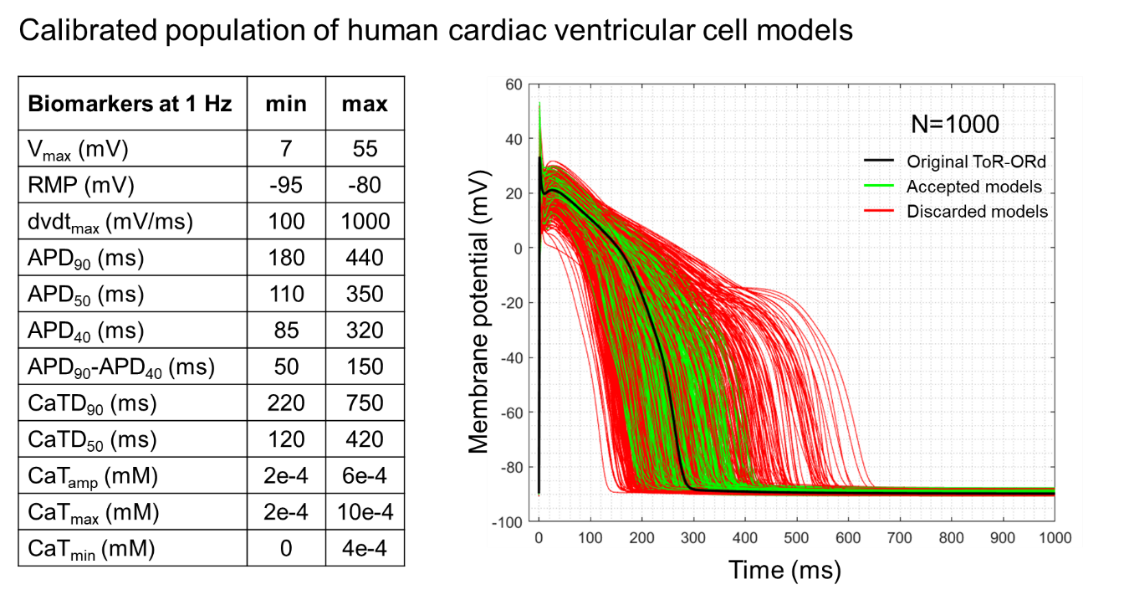


Figure S1. Population of models based on the ToR-ORd action potential model of the human ventricular cardiomyocyte and its calibration with human experimental data. Left: electrophysiological single-cell biomarkers and the range of values reported in healthy ventricular cardiomyocytes (1). Right: action potential traces of the 1000 ToR-ORd model versions: the original model is represented by a black line; accepted models (green lines) present biomarkers within the value ranges in the table, while one or more biomarkers are off the range in discarded models (red lines).

The PoM was calibrated with experimental data from human ventricular cardiomyocytes, namely biomarkers describing the calcium transient and AP morphology (1). Figure S1 (table) shows the ranges for each of the biomarkers considered for calibrating the PoM. The right panel presents the AP traces for every model in the PoM with a pacing frequency of 1 Hz. Only those models reproducing the value ranges for all biomarkers were accepted for the study (green lines, N=475), whilst the rest were discarded (red lines, N=525). As a reference, the original ToR-ORd model is represented by a black line.

Additionally, the PoM was calibrated to accept only those ToR-ORd variations (N=275) reproducing the alterations observed in ischaemia (see Figure S2).

| **Ischaemic severity** | APD_90_ experimental values in (2) | Acidosis  (I_Na_ and I_CaL_ reduction) | Hypoxia  (I_fATP_ activation) | Hyperkaelemia ([K^+^]_0_) |
| --- | --- | --- | --- | --- |
| Healthy | 206-306 ms (pre ischaemia) | 0% | 0% | 5 mM |
| Mild ischaemia | 169-293 ms  (1 min ischaemia) | 10% | 2% | 7 mM |
| Severe ischaemia | 127-291 ms  (2-3 min ischaemia) | 20% | 4% | 8 mM |

Figure S2. Calibration parameters of the PoM under ischaemic conditions. Ischaemia was simulated at different severities and compared with human experimental action potential duration (APD_90_) values at comparable ischaemia time points.

From the calibrated PoM, we selected the 17 models with the steepest slope in action potential restitution curve using the S1S2 protocol (3). In these 17 models (plus baseline ToR-ORd), the dynamic action potential restitution protocol was conducted to analyse their capability to induce alternans, a key mechanism in spiral wave induction. The model selected for conducting the VF study (ToR-ORd_VF_) presented the following features: the steepest APD obtained in the dynamic restitution protocol (Figure S3A), the highest APD difference in alternans (Figure S3B, ∆APD in ToR-ORd_VF_: 174 ms; ∆APD in baseline ToR-ORd: 10 ms) and no repolarisation abnormalities at physiological cycle lengths (350 ms), as shown in Figure S4.

Figure S5 provides details on the parameters in the ToR-ORd_VF_ model.

#
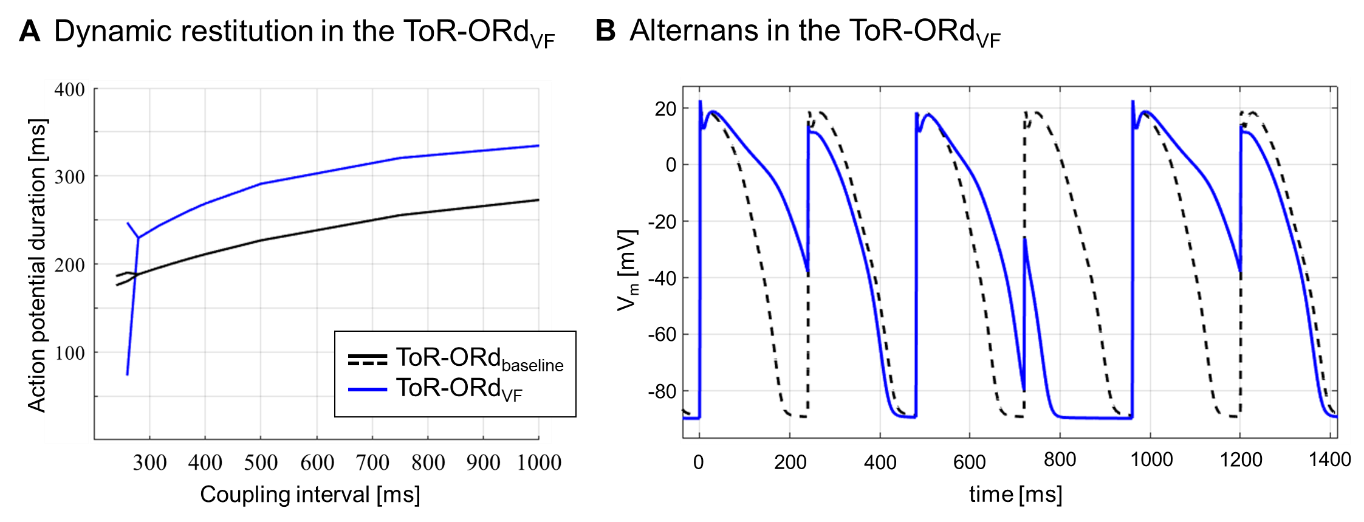


Figure S3. (A) Comparison of the dynamic action potential (AP) duration restitution curves of the baseline ToR-ORd (black) and ToR-ORd_VF_ (blue) evidencing the differences in alternans and slope steepness at short CIs. (B) Simulated APs at a short cycle length (240 ms) show the capability of the ToR-ORd_VF_ (blue) to reproduce alternans compared to the APs obtained from the baseline model in identical conditions (dashed black line).


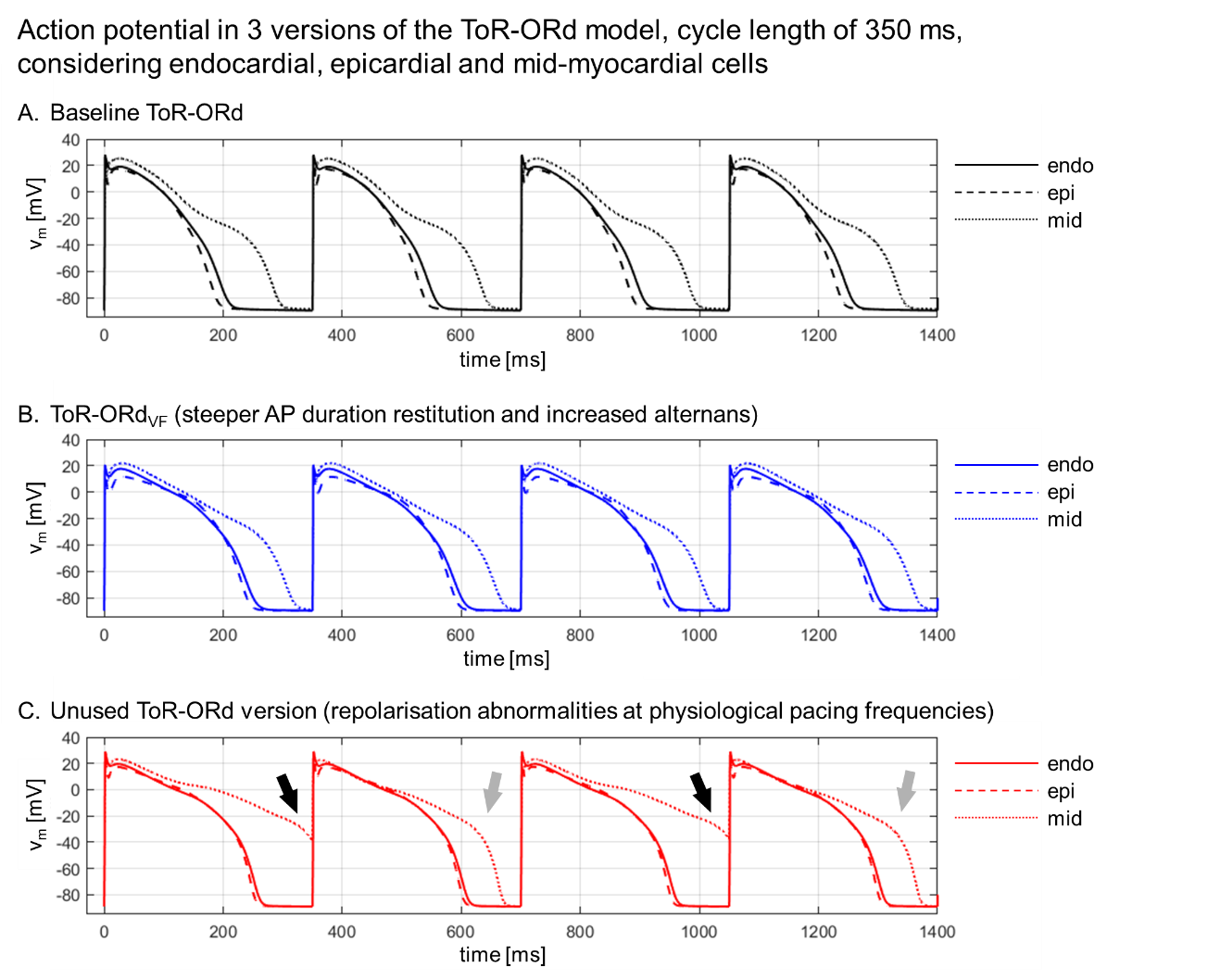


Figure S4. Action potentials (AP) obtained from endocardial, epicardial and mid-myocardial cell types at a cycle length of 350 ms for (A) the baseline ToR-ORd model, (B) the ToR-ORd_VF­_ used for the study, and (C) an unused version of the ToR-ORd presenting repolarisation abnormalities. The arrows show very pronounced alternans hampering cell repolarisation at physiological pacing frequencies.

| **ICaL** | 0.59560 |
| --- | --- |
| **INa** | 1.39239 |
| **Ito** | 1.47548 |
| **INaL** | 1.39139 |
| **IKr** | 1.13413 |
| **IKs** | 0.81782 |
| **IK1** | 0.74975 |
| **IKb** | 0.90691 |
| **INaCa** | 0.90891 |
| **INaK** | 1.27628 |
| **INab** | 1.31131 |
| **ICab** | 1.35736 |
| **IpCa** | 1.10210 |
| **ICaCl** | 0.74474 |
| **IClb** | 0.67768 |
| **Jrel** | 0.61962 |
| **Jup** | 1.36737 |
| **inact_ICaL (τ_f,fast_ and τ_f,slow_)** | 1.90103 |
| **inact_INa (τ_h_ and τ_j_)** | 0.50313 |
| **inact_NaL (τ_h,L_)** | 0.65903 |
| **inact_IKrVar8 (β_i_)** | 0.35248 |

**Figure S5.** Scaling factors applied to baseline ToR-ORd cell model parameters to obtain the model variation with a steeper action potential duration restitution used in this study (ToR-ORd_VF_).

# Additional figure


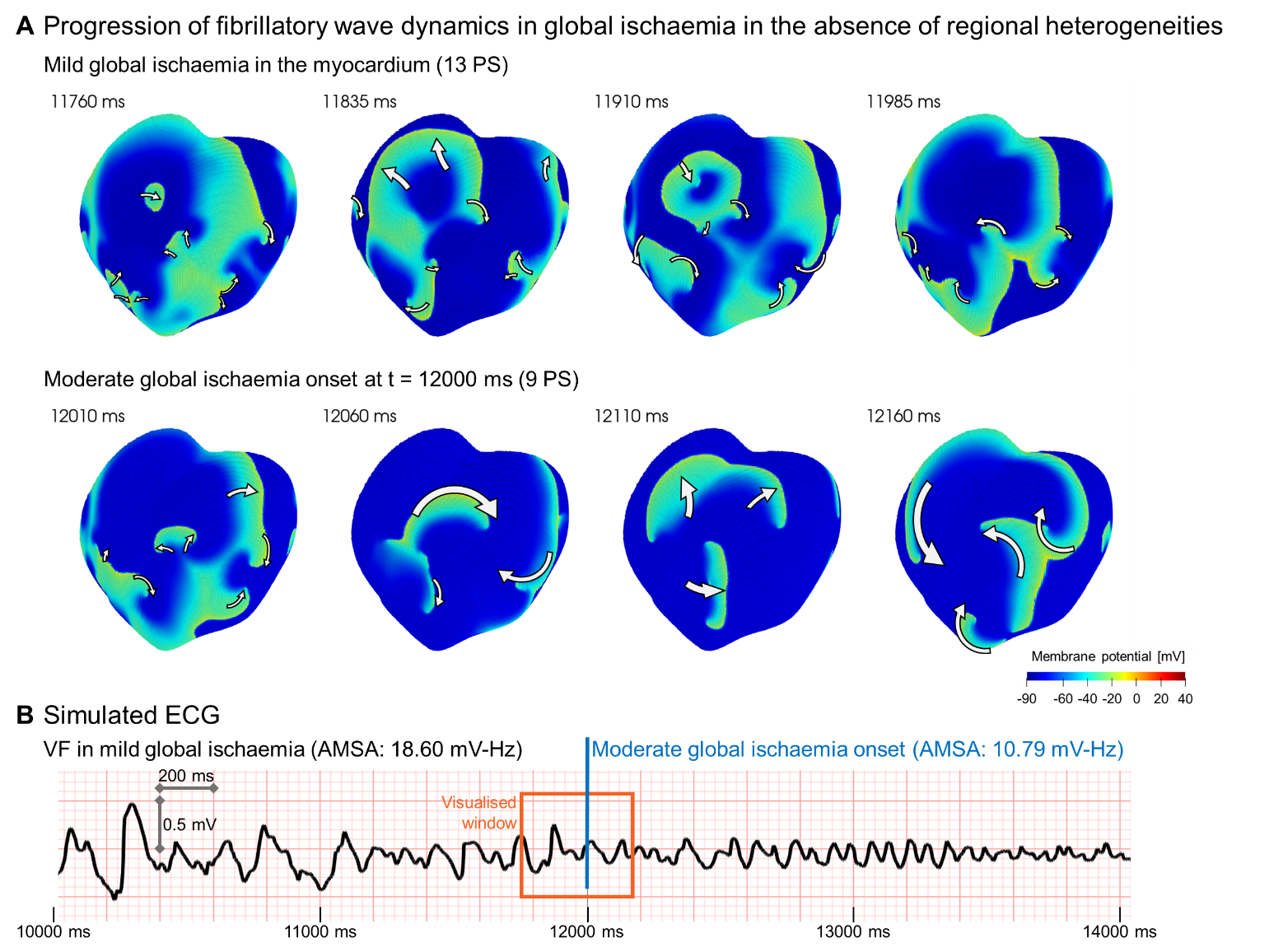


Figure S6. Progression of ventricular fibrillation (VF) dynamics under global ischaemia conditions (in the absence of regional heterogeneities) with increasing severity. A: Membrane potentials maps for mild global ischaemia (first row, t = 11760-11985 ms) and moderate global ischaemia (second row, t = 12010-12160 ms, onset at t = 12000 ms). B: Simulated ECG signal for that simulation. The red rectangle represents the visualised time window in the top panel. Annotations describe the severity of global ischaemia and computed Amplitude Spectrum Area (AMSA) values. ECG marker values based on apex-anterior electrode configuration.

# References

1. Coppini R, Ferrantini C, Yao L, Fan P, Del Lungo M, Stillitano F, et al. Late Sodium Current Inhibition Reverses Electromechanical Dysfunction in Human Hypertrophic Cardiomyopathy. Circulation. 2013 Feb 5;127(5):575–84.

2. Sutton P, Taggart P, Opthof T, Coronel R, Trimlett R, Pugsley W, et al. Repolarisation and refractoriness during early ischaemia in humans. Heart. 2000 Oct;84(4):365–9.

3. TSE G, WONG ST, TSE V, YEO JM. Restitution analysis of alternans using dynamic pacing and its comparison with S1S2 restitution in heptanol-treated, hypokalaemic Langendorff-perfused mouse hearts. Biomed Rep. 2016 Jun;4(6):673–80.
